# Influence of Exercise on Proteolysis-Related Markers of Cardiac, Renal, and Endothelial Dysfunction

**DOI:** 10.7759/cureus.108088

**Published:** 2026-05-01

**Authors:** Tai G Metzger, Joon Sung, Paul Megee

**Affiliations:** 1 Foundational Medical Studies, Oakland University William Beaumont School of Medicine, Rochester, USA

**Keywords:** exercise science, metabolomics, methylarginines, molecular biology, molecular transducers of physical activity consortium, protein degradation, proteolysis, proteomics, rat lung, ubiquitin proteasome pathway

## Abstract

Introduction: The effects of aerobic physical activity on proteolysis-related markers of cardiovascular, kidney, and endothelial dysfunction, such as asymmetric dimethylarginine (ADMA) and symmetric dimethylarginine (SDMA), have not been well studied. Our goal was to investigate the correlation between exercise and the levels of proteolysis-related markers by examining the production of ADMA and SDMA across multiple tissues and between sexes.

Methods: We analyzed publicly available data from the Molecular Transducers of Physical Activity Consortium (MoTrPAC), which included previously collected multi-omic profiling of Fischer 344 rats after progressive treadmill training. Analysis of ADMA/SDMA was performed using untargeted hydrophilic interaction liquid chromatography* *(HILIC)-positive mass spectrometry. Gene expression of ADMA/SDMA producers (protein arginine methyltransferases (PRMTs)) and proteasome-related *PSMC *genes was examined via RNA-seq in skeletal muscle (gastrocnemius), kidney, liver, adipose tissue, plasma, heart, and small intestine tissue. In cohorts of 50 or 60 mice, data were analyzed over one, two, four, and eight weeks post training and compared to sedentary controls.

Results: Composite ADMA/SDMA levels in brown adipose rose significantly in females and to some extent in males. ADMA and SDMA levels decreased in the small intestine in both sexes, while kidney SDMA rose in males and slightly in females. SDMA also increased transiently in female skeletal muscle. RNA-seq revealed sex-specific changes in proteasome-related *PSMC* gene expression, including upregulation in female kidney and downregulation in male liver and adipose tissue. PRMT expression decreased in male blood, and PRMT 1-3 trended upward in male brown adipose.

Conclusion: These findings suggest that exercise induces complex tissue- and sex-specific patterns of proteolysis and methylarginine regulation, showing potential associations related to the metabolic benefits of physical activity.

## Introduction

Sedentary lifestyle and obesity are associated with numerous negative health effects, such as diabetes, cardiovascular disease, and kidney disease, while exercise has many positive benefits [1]. Identifying the molecular mechanisms behind these effects could allow for identification of therapeutic targets and early markers of disease states [2]. Protein degradation is important for the clearance of defective proteins, regulation of protein levels to meet bodily needs, and production of amino acid precursors for metabolic processes [3-5]. A prior study has also shown that exercise-induced changes were not identical across all tissues and that sex differences existed [6]. We hypothesize that one of the mechanisms by which exercise promotes improved health could be by influencing ordered proteolysis pathways. 

Previous research has shown the important beneficial effects of protein degradation followed by protein synthesis in skeletal muscle following exercise [7,8]. Exercise increases rates of protein degradation in the skeletal muscle as well as net protein degradation according to whole-body measurements [9-11]. In contrast, obesity has been shown to interfere with the beneficial effects of exercise in skeletal muscle [12,13]. 

While the positive effects of proteolysis due to exercise have been well studied in skeletal muscle, there has been less investigation into these effects in other tissue types. There have been conflicting findings regarding the effects of exercise on proteolysis in the liver. One study found that endurance exercise increased lysosomal autophagy in the liver [14]. However, another study found that acute exercise increased liver proteolysis in rats but not in dogs [15]. Thus, there exists a gap in the literature regarding the effect of exercise on proteolysis in organs other than skeletal muscle. We were also interested in studying these effects in both brown and white adipose tissue due to their contrasting effects on metabolic health. White adipose tissue (WAT) primarily stores energy and functions as an endocrine organ regulating appetite, insulin sensitivity, and inflammation, whereas brown adipose tissue (BAT) dissipates energy through non-shivering thermogenesis. BAT activation improves glucose and lipid metabolism, offering protective effects against obesity and metabolic diseases [16].

Proteolysis mainly occurs through two pathways: the ubiquitin proteasome pathway (UPP) and the lysosomal pathway [5]. We hypothesized that asymmetric dimethylarginine (ADMA) and symmetric dimethylarginine (SDMA), which are byproducts of proteolysis, may be involved in mediating the effects of exercise on proteolysis. ADMA and SDMA are modified amino acids produced during normal protein turnover by protein arginine methyltransferases (PRMTs) [17]. However, when ADMA and SDMA accumulate in the body, they are potent endogenous inhibitors of nitric oxide synthase (NOS) and are markers of cardiovascular disease, endothelial dysfunction, pre-diabetes, and kidney disease [17-19].

Prior work mapping molecular responses to endurance training has been biased toward male animals, limiting understanding of how biological sex affects exercise adaptations [20,21]. Recent multi-omic studies have shown that exercise has distinct physiological and molecular effects between males and females, specifically in metabolic, inflammatory, and mitochondrial responses to training across multiple tissues [22-24].

In the Molecular Transducers of Physical Activity Consortium (MoTrPAC) study, after eight weeks of training, male rats showed a 5% decrease in body fat, while female rats showed no change in body fat percentage [6]. The body weight for female rats increased in all intervention groups, while male rats showed no changes. In the adrenal gland, endurance training impacted a large cluster of genes involved in steroid hormone biosynthesis and energy metabolism, including transcripts regulated by PPARa, PPARy, and ERRy, which showed opposite temporal patterns between sexes, with persistent down-regulation in females and an early, yet transient increase in males. In the rat lung, endurance training decreased phosphosignaling activity more in males than in females. The largest sex difference came from the protein kinase cAMP-activated catalytic subunit alpha (PRKACA) pathway, a kinase that participates in many cellular processes. Four cellular structure-associated PRKACA substrates, DSP, MYLK, STMN1, and SYNE1, similarly decreased in activity. Protein phosphorylation within the lung suggests a sex-dependent role for the PRKACA pathway in regulating lung structure and function with endurance training. Male rats, but not female rats, showed strong activation of immune pathways and transcription factors in both white and brown adipose tissue with endurance training. In the small intestine, female rats showed robust down-regulation of immune-related transcripts at eight weeks of endurance training, dampening genes with known inflammatory bowel disease genetic risks such as *MHCII*, *Cxcr3*, and *Il1a* genes [6].

These studies highlight sex as a key biological variable that can affect how proteolysis and related signaling pathways respond to endurance training, but did not directly investigate whether sex differences contribute to observed changes in exercise-induced proteolysis. Therefore, comparing both sexes is essential for identifying sex-specific mechanisms by which endurance training may impact proteolysis and methylarginine production. 

The effects of regular exercise on protein degradation and ADMA/SDMA production in other tissues, such as the liver, kidney, heart, small intestine, and adipose tissue, remain unclear. The objective of this study was to determine the effects of exercise on protein degradation pathways and ADMA/SDMA production across multiple tissues. The primary independent variable in our study was exercise time, specifically, a progressive treadmill training program. Sex was also studied as an independent variable. The dependent variables were the amount of metabolite, gene expression, and protein levels of the molecules of interest related to proteolysis and ADMA/SDMA production. We hypothesized that endurance training may mediate some of its beneficial effects by influencing proteolysis and ADMA/SDMA production in various tissues, consequently influencing metabolic dysfunction and endothelial risk. 

## Materials and methods

This study comprised an analysis of publicly available data from the MoTrPAC Data Hub (https://motrpac-data.org/) [2]. This data was previously collected from six-month-old male and female Fischer 344 rats utilizing multi-omic analysis of whole blood, plasma, and 18 solid tissue types. The Fischer 344 rats were chosen because they are a well-characterized strain known to develop age-associated insulin resistance, adiposity, and ectopic lipid deposition, which represent disease processes that endurance exercise has been shown to improve [25,26]. Multi-omic analysis consisted of genomics, proteomics, metabolomics, and protein immunoassay technologies. This data collection occurred at one, two, four, and eight weeks following initiation of a progressive treadmill training protocol meant to simulate endurance training and was compared to data collected from sedentary rats, which served as the control group [6]. A total of 50 rats, including five male and five female rats per timepoint, were used for RNA-seq, and 60 rats, including six male and six female rats per timepoint, were used for proteomics [6,27].

Exercise protocol

The exercise protocol is summarized from the MoTrPAC study, in which Fischer 344 rats underwent an eight-week, progressive training protocol on a treadmill [2]. The rats were acclimated to the treadmill over 12 days, and the rats that successfully completed the familiarization protocol were randomly assigned to a control or training group to ensure that the mean body weights of the groups were equal. Rats exercised five days per week on a five-lane motorized treadmill at an intensity of approximately 70% of maximal oxygen consumption (VO2 Max). Training began at a 5° incline for 20 minutes at 13 meters/minute for males and 16 meters/minute for females. Training duration increased by one minute per day until reaching 50 minutes by week seven. The treadmill incline was raised to 10° at week three through the remainder of the training period. Treadmill speed was increased at the start of weeks two, four, five, six, and seven, reaching a peak at 25 meters/minute for males and 28 meters/minute for females. These sex-specific protocols were chosen so that males and females exercised at a comparable relative intensity of approximately 70% of maximal oxygen consumption (VO2 Max), based on pre-training VO2 max measurements and an established intensity-controlled treadmill protocol [6,28]. Control rats were placed on a stationary treadmill (0 meters/minute) for 15 minutes per day and five days per week. All tissue collection occurred 48 hours after the final exercise session to minimize acute training effects. 

Data collection

For metabolite analysis of ADMA/SDMA levels, we examined data collected using the untargeted HILIC-positive assay. Untargeted HILIC-Positive is a metabolomics technique that uses hydrophilic interaction liquid chromatography (HILIC) coupled with mass spectrometry to identify and quantify a range of metabolites in a sample of unknown compounds [29]. This allowed for a comprehensive analysis of the metabolome [2]. We first analyzed composite ADMA/SDMA levels in brown adipose tissue measured using the untargeted HILIC-positive technique. We examined the composite ADMA/SDMA levels for brown adipose tissue because data for ADMA/SDMA separately were not available. We then analyzed ADMA/SDMA untargeted HILIC-positive levels individually in skeletal muscle (gastrocnemius), kidney, liver, plasma, heart, and small intestine tissue, as well as white and brown adipose tissue.

For analysis of gene expression, we examined data collected using RNA sequencing (RNA-seq). RNA-seq is a high-throughput technique used to analyze the transcriptome, the set of RNA transcripts expressed by the genome under specific conditions. The process involves isolating RNA, converting it to complementary DNA (cDNA), fragmenting and tagging it with sequencing adapters, and then sequencing the fragments using next-generation sequencing (NGS) platforms. This allows for quantitative and qualitative assessment of gene expression. We examined RNA-seq LogFC (fold change) levels for each of these genes at one, two, four, and eight weeks in skeletal muscle (gastrocnemius), kidney, liver, white adipose, plasma, heart, small intestine, and brown adipose tissues. 

Proteomic analyses focused on proteins involved in ADMA/SDMA metabolism, including protein arginine methyltransferases (PRMTs), as well as subunits of the proteasome. These targets were selected based on their established roles in methylarginine production and protein degradation pathways, and analyses were limited to proteins available within the MoTrPAC proteomics dataset. Data was collected as LogFC, representing the log of the ratio in concentration at one, two, four, and eight weeks relative to sedentary control rats. Details regarding the methodology outlined here are based on the information that is publicly available on the MoTrPAC Data Hub and previous publications [2,6]. For more information, readers are invited to see the supplemental materials of these publications [2,6], which contain extensive details regarding how the data were collected.

Statistical analysis

RNA-seq Data Normalization

RNA sequencing and analysis methods are described in detail in the Supplemental Information of previous MoTrPAC publications [2,6]. To summarize, RNA sequencing libraries were generated and sequenced on an Illumina NovaSeq 6000 platform (Illumina, Inc., San Diego, California, United States) to a target depth of ~70 million paired-end reads per sample. Raw reads underwent quality control, adapter trimming, and filtering of low-quality sequences before alignment to the Ensembl *Rattus norvegicus* (rn6) reference genome using STAR (Spliced Transcripts Alignment to a Reference). Gene-level expression was quantified with RSEM (RNA-Seq by Expectation-Maximization), and unique molecular identifiers (UMIs) were used to account for polymerase chain reaction (PCR) duplicates. Lowly expressed genes were filtered, and normalized expression values were generated using the trimmed mean of M-values (TMM) normalization followed by log-transformed counts per million. Differential expression analysis was performed using DESeq2, which applies a negative binomial modeling framework to identify genes with statistically significant differences in expression between conditions while accounting for dispersion and library size differences [2,6]. 

Proteomics Data Normalization

Proteomics data were quantified using log2-transformed tandem mass tag (TMT) ratios relative to a common reference. Quality control included identification and removal of outlier samples and contaminant features, as well as exclusion of poorly quantified proteins. Data were normalized by median-centering and mean absolute deviation scaling, and batch effects across TMT plexes were corrected using linear models implemented in limma. Post-translational modification (PTM) datasets were adjusted for underlying protein abundance using global linear modeling; however, protein-corrected PTM values were only applied in select analyses due to incomplete overlap with total proteome measurements [2,6]. 

Statistical Tests

Statistical significance was defined as alpha less than 0.05 for the difference between the exercise data point and the sedentary control rats using the Analysis of Variance (ANOVA) statistical test. This test was performed to assess for statistically significant differences between the average value of the dependent variable (e.g., level of the metabolite or gene product being studied) in the exercise rats at that time point (i.e., one, two, four, eight weeks) compared to the average value of the sedentary control rats at the same time point. Thus, statistical significance indicated that the molecule in question was significantly increased or decreased after the stated number of weeks of training compared to the levels of the molecule being studied in the control rats. All data was entered into Google Sheets 2025 web version (Google LLC, Mountain View, California, United States) to produce results tables and figures. Sciex OS (AB SCIEX, Version 1.6.1; Released 2012; Sciex, Marlborough, Massachusetts, United States) was used for statistical analysis of mass spectrometry data by the original MoTrPAC study group.

## Results

**Figure 1:**
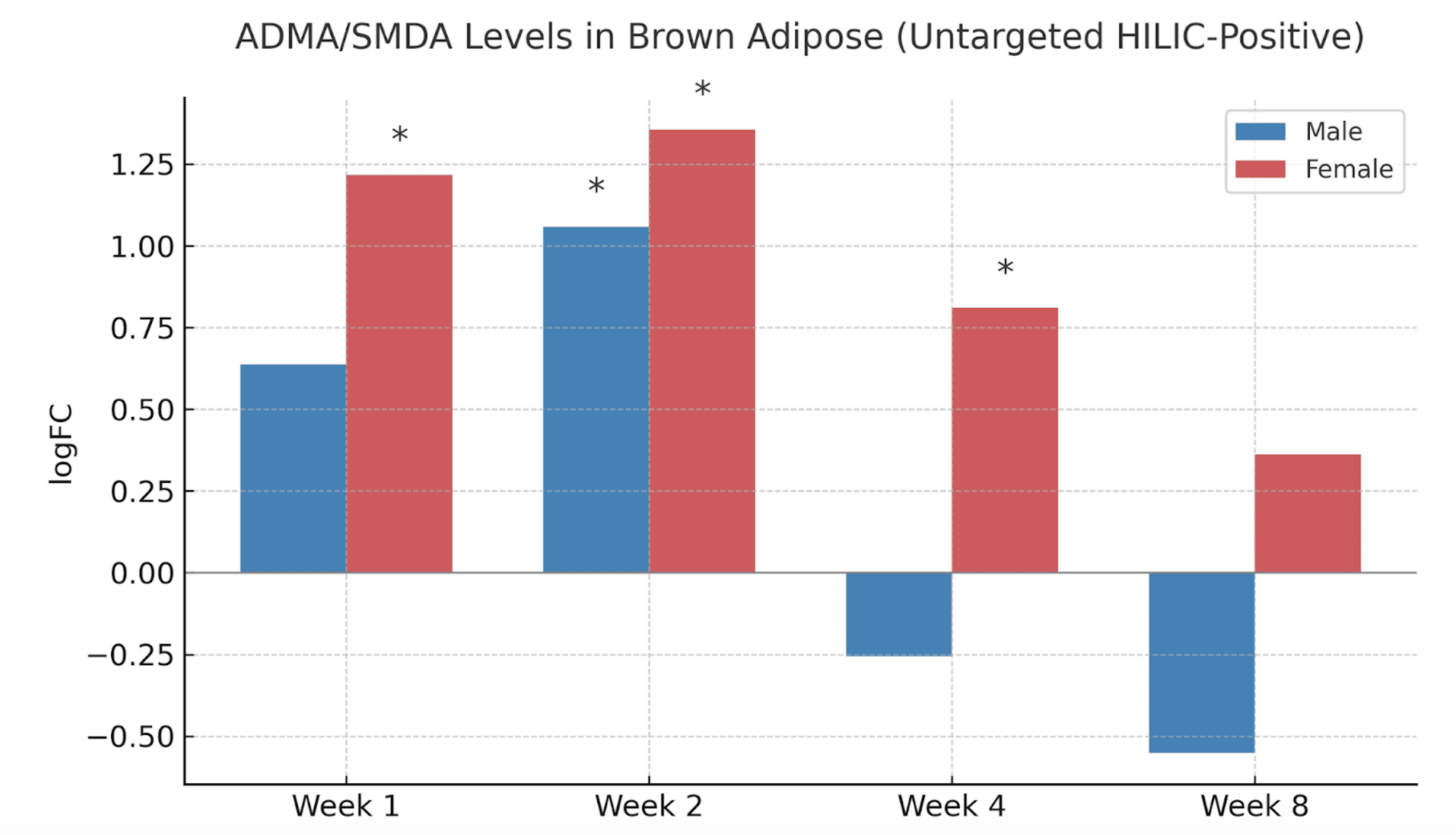
Composite ADMA/SMDA log fold changes in brown adipose following training. Asterisks indicate p<0.05 for comparison to corresponding sedentary control rats. ADMA: asymmetric dimethylarginine; SDMA: symmetric dimethylarginine; HILIC: hydrophilic interaction liquid chromatography

Following endurance training, composite ADMA/SDMA levels in brown adipose tissue exhibited sex-specific and time-dependent trends. In female rats, there was a significant increase in logFC values at weeks one, two, and four, indicating elevated ADMA/SDMA production during the early and intermediate phases of training. In contrast, male rats showed a significant increase only at week two, with levels declining at weeks four and eight (Figure 1).

**Figure 2:**
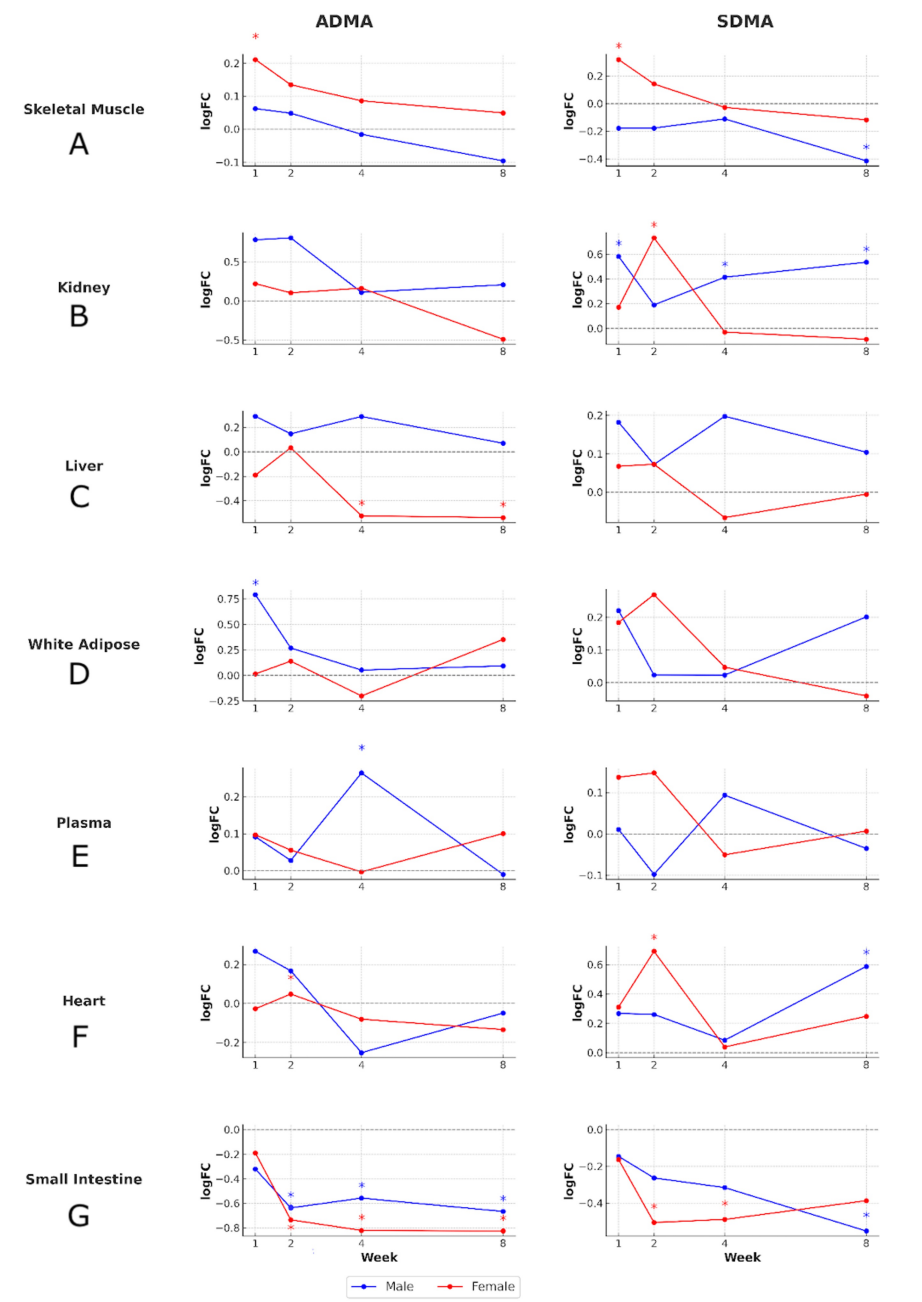
Individual log fold changes of ADMA and SDMA in various tissues following training (untargeted HILIC-positive). Asterisks indicate p<0.05 for comparison to corresponding sedentary control rats. ADMA: asymmetric dimethylarginine; SDMA: symmetric dimethylarginine; HILIC: hydrophilic interaction liquid chromatography

Levels of ADMA demonstrated distinct tissue- and sex-specific responses to exercise (Figure 2). Notably, skeletal muscle ADMA significantly increased after one week of training in female rats, while no such increase was observed in male rats during this period. In the liver, ADMA levels in female rats decreased significantly at both weeks four and eight. In male rats, WAT showed a significant increase in ADMA at week one, and plasma levels also rose significantly at week four. Both male and female small intestine tissues exhibited a consistent and significant reduction in ADMA levels at weeks two, four, and eight, highlighting this tissue as a potential site of suppressed ADMA-mediated signaling during prolonged training.

The SDMA profiles further illustrate tissue- and sex-specific adaptations (Figure 2). In female rats, SDMA increased significantly in skeletal muscle at week one and in the heart at week two. In the kidney, female rats exhibited a significant increase at week two, while male rats showed sustained elevations at weeks one, four, and eight. Conversely, SDMA levels in the small intestine decreased significantly in female rats at weeks two and four, and in male rats at week eight.

RNA-seq for PRMTs and proteasome genes

Next, we analyzed RNA-seq data for genes involved in ADMA/SDMA production (PRMTs) and genes of the UPP Proteasome (*PSMC*). Specifically, type I PRMTs (PRMT1, PRMT3, PRMT4, PRMT6) primarily produce ADMA, type II PRMTs (PRMT5, PRMT9) generate SDMA, and type II PRMTs (PRMT7) produce monomethylarginine. The *PSMC* genes (Proteasome 26S Subunit, ATPase) are a family of genes (*PSMC1*, *PSMC2*, *PSMC3*, etc.) that play a crucial role in protein degradation within cells. Along with Sem1, which plays a crucial role in the stability and assembly of the 26S proteasome, the PSMC proteins are essential components of the 26S proteasome complex required for the UPP. 

RNA-seq data for proteasome-related* PSMC *genes revealed significant sex- and tissue-specific expression patterns (Table 1). In females, *PSMC1* expression increased notably in the kidney, alongside general elevations in other *PSMC *genes in this tissue, although not all changes reached statistical significance. In contrast, male rats exhibited widespread and significant downregulation of* PSMC *genes after eight weeks, particularly in the liver and white adipose tissue. Additionally, several *PSMC* genes were significantly downregulated in male BAT, while female rats demonstrated reductions in PSMC expression in skeletal muscle and WAT, highlighting divergent tissue-specific responses in proteasome activity between sexes. There were also some increases in *PSMC* in the female small intestine tissue. 

**Table 1:** Proteasome subunit mRNA levels following training (RNA-seq) LogFC values of RNA-seq for proteasome genes after one, two, four, and eight weeks of training. Asterisk indicates p-value < 0.05.

Genes of the Ubiquitin Proteasome Pathway (UPP): RNA-seq																	
LogFC		Skeletal Muscle		Kidney		Liver		White Adipose		Blood RNA		Heart		Small Intestine		Brown Adipose	
		Male	Female	Male	Female	Male	Female	Male	Female	Male	Female	Male	Female	Male	Female	Male	Female
PSMC1	Week: 1	0.0237	-0.0464	-0.0252	*0.0962	-0.0194	-0.0084	-0.0023	-0.1126	-0.1567	-0.1274	-0.035	0.0271	0.0213	*0.1721	-0.11	-0.0799
	2	0.0399	0.0593	0.0229	0.051	-0.0324	-0.0241	-0.0304	-0.0783	*-0.4074	0.0122	0.0389	*0.0839	-0.0235	0.1326	-0.1172	-0.1729
	4	0.041	0.0233	-0.0144	*0.0736	0.0052	-0.0019	0.0701	-0.0258	0.0895	-0.1373	0.0057	0.0707	0.0091	0.1016	-0.1782	-0.0437
	8	0.0124	-0.031	-0.0539	*0.0808	*-0.1425	-0.0049	-0.0059	0.029	-0.3037	-0.0209	-0.0556	0.0015	0.0012	0.0724	*-0.3387	-0.205
PSMC2	Week: 1	-0.0091	0.0066	0.089	*0.1304	-0.0633	-0.007	-0.0381	0.0146	0.0925	-0.2018	0.0517	0.0391	0.0402	*0.1593	-0.1359	0.026
	2	0.0599	0.0528	0.0748	0.0897	-0.0979	-0.0125	-0.0697	0.178	-0.0647	0.0371	0.0739	*0.0753	0.0191	0.0474	-0.0485	-0.0051
	4	0.0319	0.0139	0.0209	0.0429	-0.1128	0.0776	-0.0204	0.0866	0.0897	0.0352	0.0511	0.0578	-0.0065	0.1221	-0.0865	-0.0025
	8	0.0211	-0.0216	0.0637	-0.0117	*-0.2001	-0.0641	*-0.125	0.1289	0.1006	-0.1575	-0.063	0.0165	-0.0148	0.0465	-0.2249	-0.155
PSMC3	Week: 1	-0.0203	*-0.1052	0.0351	0.0534	-0.0594	0.0324	*-0.2153	-0.0197	-0.0862	*-0.3168	0.0025	-0.0068	-0.0348	0.0607	-0.0506	0.109
	2	0.0061	-0.0327	0.0567	0.0774	-0.0031	0.0024	*-0.1804	0.1465	-0.0931	-0.0474	0.0218	0.0148	-0.0686	0.0513	-0.0373	-0.0194
	4	0.0383	-0.0056	0.0405	0.0361	-0.0984	0.0726	-0.0421	0.0367	0.045	-0.1217	-0.0036	0.0344	-0.0027	-0.0312	0.0055	-0.0419
	8	0.0242	*-0.1097	0.0556	0.0354	*-0.1422	0.0688	-0.0808	0.1613	0.0302	-0.0122	-0.0039	-0.0203	-0.0206	0.0638	-0.0449	0.0091
PSMC4	Week: 1	-0.0676	-0.0379	-0.0486	0.0775	-0.0629	-0.0456	-0.0776	0.0431	-0.0044	-0.1515	-0.0124	-0.0509	-0.0275	0.0548	-0.0831	-0.01
	2	-0.0087	-0.0317	0.0711	0.0473	-0.1123	-0.0644	-0.0736	0.0944	-0.0065	-0.1264	-0.0069	0.0319	-0.0605	0.0385	-0.146	-0.1207
	4	-0.0531	-0.0608	-0.002	-0.0024	-0.1324	0.0272	-0.0553	0.0079	0.0772	-0.0728	-0.007	-0.0653	-0.0328	0.0071	-0.2188	0.0137
	8	-0.0187	-0.068	-0.0809	0.0229	*-0.3201	-0.094	*-0.1176	0.0161	0.1304	-0.0918	-0.0669	-0.0706	-0.0247	-0.0372	*-0.4215	-0.1411
PSMC5	Week: 1	-0.0036	-0.0833	0.0566	*0.1254	-0.0344	-0.0787	-0.0481	-0.0278	-0.1291	-0.0636	0.0431	-0.0198	-0.0433	0.0439	-0.1883	-0.0401
	2	0.0438	-0.0125	-0.0113	0.1072	-0.0727	-0.1124	-0.1272	0.0042	-0.2273	0.0839	0.0428	0.0527	-0.0806	-0.0294	-0.2759	0.0106
	4	*0.0941	0.0351	-0.0195	0.0017	-0.0944	0.002	-0.022	0.0687	0.043	0.0652	0.0097	0.0388	-0.074	-0.1553	-0.1549	-0.0787
	8	0.0248	-0.066	-0.0081	0.0448	*-0.2152	-0.0973	*-0.1938	0.1337	0.0853	-0.0569	*-0.0851	-0.0476	-0.0572	-0.0094	*-0.6086	-0.2399
SEM1	Week: 1	0.0149	*-0.1603	0.0073	0.1451	-0.0179	0.0356	-0.1667	-0.0581	0.1487	-0.0192	0.0309	*-0.117	-0.068	0.0337	-0.1281	0.0547
	2	0.0947	-0.0791	0.0592	0.0569	-0.0411	0.0617	-0.1684	0.0528	0.1355	-0.0902	0.0186	*-0.1189	0.0315	0.0318	-0.1513	0.075
	4	0.0872	-0.0922	-0.0123	0.0234	-0.0272	0.1472	-0.0451	0.0581	0.1964	-0.106	-0.008	-0.0302	-0.0261	-0.0346	-0.2005	0.1611
	8	0.0177	-0.1278	0.0398	0.0819	-0.1412	0.0582	-0.0594	*0.2151	0.3626	-0.0933	0.0301	-0.0275	-0.084	0.0584	*-0.4454	-0.0476

*PRMT* gene expression patterns, which influence ADMA and SDMA production, showed broad suppression in specific tissues (Table 2). In male blood RNA, there were consistent and significant decreases in nearly all *PRMT*s following training after eight weeks. *PRMT1* levels trended downward in both male and female heart tissue. In male brown adipose tissue, *PRMT* 1-3, responsible for ADMA synthesis, displayed increased expression trends. In contrast, female WAT showed significant increases in *PRMT7* and *PRMT9* at week eight, while male WAT demonstrated *PRMT* downregulation, reaching significance for *PRMT5* and *PRMT7*. 

**Table 2:** PRMT mRNA levels following training (RNA-seq) LogFC values of RNA-seq for ADMA/SDMA-producing genes after one, two, four, and eight weeks of training. Asterisk indicates p-value < 0.05. ADMA: asymmetric dimethylarginine; SDMA: symmetric dimethylarginine

PRMTs (produce ADMA/SDMA): RNA-seq																	
LogFC		Skeletal Muscle		Kidney		Liver		White Adipose		Blood RNA		Heart		Small Intestine		Brown Adipose	
		Male	Femal	Male	Female	Male	Female	Male	Female	Male	Female	Male	Female	Male	Female	Male	Female
PRMT1	Week: 1	-0.0235	*-0.2196	-0.083	0.0839	-0.2199	0.1477	0.1054	0.1723	-0.26	-0.0262	-0.1225	-0.0847	-0.1105	-0.1285	0.2396	0.1461
	2	-0.0303	*-0.1628	-0.0795	0.067	-0.2667	-0.0292	-0.0414	0.2947	-0.1191	-0.3548	*-0.1372	*-0.1372	-0.1413	0.0012	-0.0385	-0.0335
	4	0.0023	-0.047	-0.0773	0.0443	-0.2936	-0.0045	0.0186	0.087	0.0095	-0.3833	-0.0942	-0.1084	-0.0002	-0.0751	0.2084	0.0352
	8	-0.0835	-0.1146	-0.0663	0.0476	-0.2058	-0.0503	-0.0166	0.2278	-0.2844	-0.2882	-0.1037	*-0.1903	-0.0714	0.0981	0.2128	0.0517
PRMT2	Week: 1	-0.3189	0.1973	0.0301	0.0064	-0.1737	-0.0105	0.1279	-0.2055	-0.182	-0.4772	-0.0301	-0.0189	-0.0441	*0.3843	0.284	-0.179
	2	-0.1298	0.0846	-0.1787	0.0878	0.0078	0.3751	-0.0162	-0.1523	-0.117	-0.0239	-0.1194	-0.0457	-0.0587	0.1425	0.1186	-0.2016
	4	-0.154	0.067	-0.0618	0.0257	0.0358	0.3195	-0.0173	-0.2247	0.0942	-0.2891	0.0044	0.0835	-0.1741	0.1366	0.4099	-0.2685
	8	-0.3376	0.0788	0.1219	0.0184	*0.6478	-0.2943	-0.1594	-0.1533	-0.3611	*-0.4188	0.0109	-0.1152	-0.0654	-0.0236	*1.0502	-0.0157
PRMT3	Week: 1	0.0702	0.0807	-0.0574	0.0213	0.0835	0.0643	0.1198	0.3169	-0.3438	0.0871	*0.2332	-0.0075	-0.0042	-0.1579	0.2626	0.0731
	2	0.1178	0.144	-0.1135	-0.0842	0.1242	-0.0727	-0.0104	0.1611	-0.4186	0.2713	0.1597	-0.0199	-0.0262	-0.1096	0.0104	0.1355
	4	0.1016	0.0172	-0.0059	-0.0312	-0.0619	-0.0919	0.0685	0.0672	-0.2357	-0.0521	0.1726	-0.0042	0.1475	-0.1299	0.401	-0.3032
	8	0.0761	-0.0419	-0.0554	-0.0154	0.1576	-0.1808	-0.1409	0.0113	*-0.8315	0.114	0.1335	0.0947	0.0883	0.0357	0.6804	-0.1918
CARM1 (PRMT4)	Week: 1	-0.0124	0.0954	0.0184	0.0947	-0.0064	0.0128	0.0373	0.0302	-0.2678	-0.022	0.0458	-0.0268	-0.0699	*0.1717	0.1328	0.1084
	2	0.0649	0.0516	-0.0004	0.0936	0.0006	-0.0391	0.0248	0.0758	-0.3491	-0.0546	0.0654	0.0331	-0.0099	0.1227	0.0583	0.0047
	4	-0.0372	0.0061	0.0109	0.0748	0.0069	0.0229	-0.0441	0.0094	*-0.3009	0.0643	*0.109	-0.0259	-0.0029	0.0489	0.023	-0.0035
	8	-0.0635	-0.0461	-0.0538	0.0387	-0.0676	-0.0298	-0.0654	0.0345	*-0.4842	-0.1177	*0.0883	-0.03	0.0072	0.0585	-0.1406	-0.0423
PRMT5	Week: 1	0.0476	*0.1622	-0.0346	*0.1501	-0.0283	0.0895	-0.0396	0.1699	-0.2036	0.3818	0.0333	0.035	-0.044	-0.0155	0.0519	*0.272
	2	0.0601	0.0594	0.0119	-0.0335	0.0715	0.0654	*-0.1328	0.1901	-0.3787	0.1124	-0.0021	0.0776	-0.1407	0.1103	-0.1126	0.102
	4	0.0346	0.0061	-0.0727	-0.0131	-0.0806	-0.1472	-0.0966	0.1097	-0.016	0.2297	0.0617	0.0665	0.0455	-0.0515	0.0314	0.099
	8	*0.1353	0.0429	-0.0167	-0.0206	0.0804	-0.0845	-0.0948	0.1746	-0.4797	0.076	0.0105	0.0419	-0.0012	0.1331	-0.1615	0.1625
PRMT9	Week: 1	-0.0346	0.0146	-0.1261	-0.0888	-0.0547	-0.1216	0.0067	0.0777	-0.1698	-0.0568	-0.0804	0.0743	*-0.2383	-0.1626	-0.0571	-0.0406
	2	-0.0313	-0.0299	-0.0498	-0.0963	-0.0073	-0.1471	-0.0241	0.0151	-0.1247	0.0293	-0.0317	0.0891	-0.0721	0.0314	0.0608	-0.1432
	4	0.0697	0.084	0.0897	-0.0739	-0.0046	-0.092	-0.0358	0.1259	-0.1566	0.1211	-0.0293	0.1074	0.0068	-0.1136	0.0232	-0.0069
	8	0.0826	-0.0208	*0.1689	-0.0235	-0.0663	0.0291	0.0049	*0.1749	-0.1524	-0.1523	-0.05	0.0744	0.0254	0.0964	0.0497	0.0029
PRMT7	Week: 1	-0.0859	0.0883	0.1706	-0.1124	-0.0411	0.0595	-0.1683	0.1313	-0.4436	0.4294	0.1093	0.0047	-0.043	-0.196	-0.248	0.207
	2	-0.1192	-0.0375	0.1301	-0.1459	0.0593	-0.1952	-0.2358	0.1104	-0.2841	0.2619	-0.0001	0.0061	-0.1701	-0.0543	-0.0859	0.0631
	4	-0.1099	0.0385	0.0531	-0.0366	-0.0727	*-0.3285	-0.0537	0.1897	-0.1725	0.3146	0.0322	0.0416	0.0486	-0.0979	-0.1636	-0.0005
	8	-0.0829	-0.0588	0.0363	-0.0396	-0.0058	-0.0114	-0.1014	*0.253	-0.4517	0.317	-0.0214	0.0618	0.1059	0.0636	-0.1181	-0.0353

Global proteomics 

**Figure 3:**
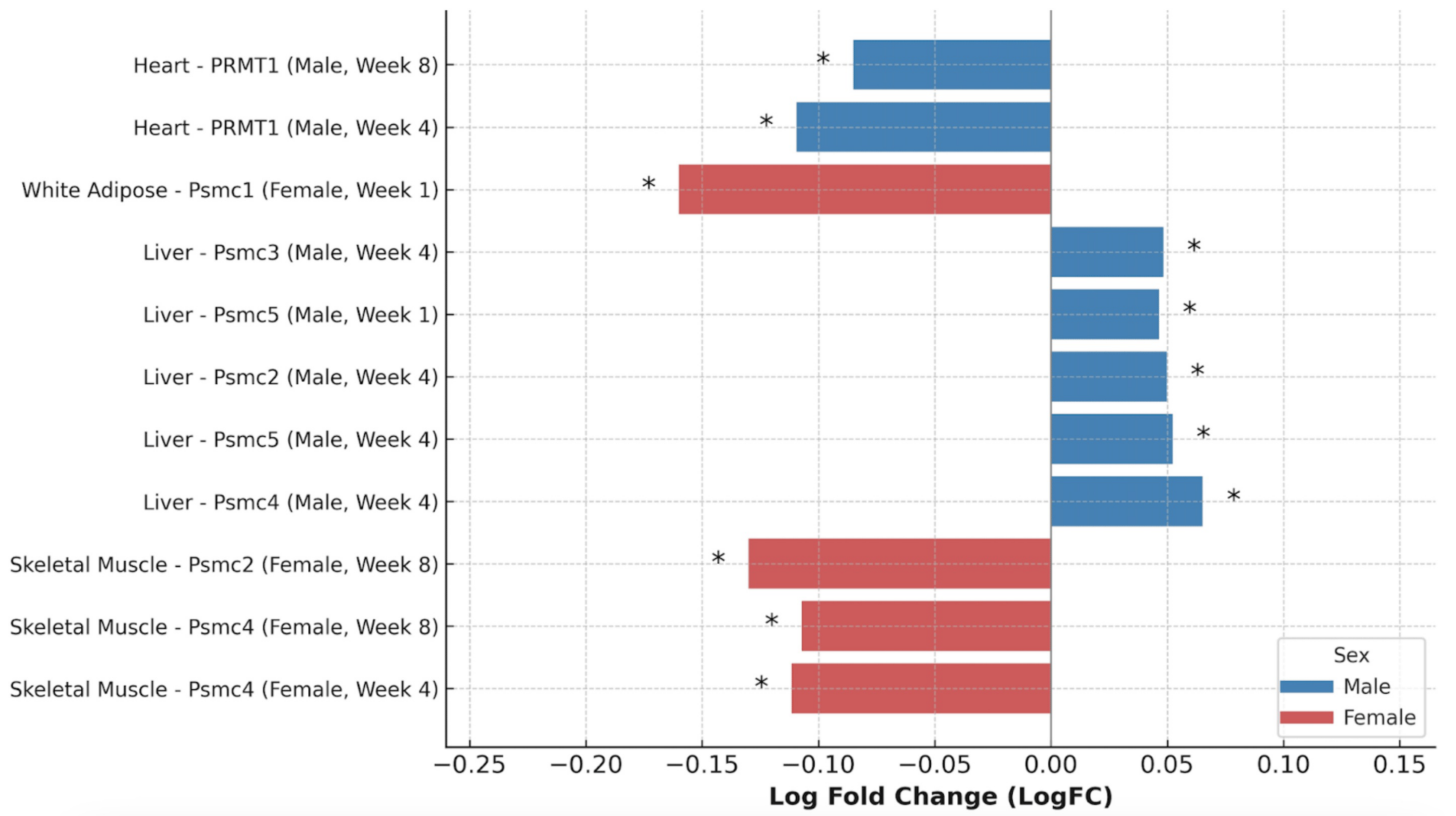
Significant changes in proteasome and PRMT global proteomics following endurance training. Asterisks indicate p<0.05 for comparison to corresponding sedentary control rats.

Based on the trends we identified in the RNA-seq data, we examined global proteomics data for notable proteins, although data for many tissues and proteins were incomplete. For PRMTs, we analyzed all PRMT family members in male blood samples and in male and female white adipose tissue. Additionally, we examined PRMT1 and PRMT2 in female blood, PRMT1 in male and female heart, PRMT1 in female skeletal muscle, and PRMT1-3 in male BAT. For UPP-related proteins, we assessed all core proteasome subunits in female skeletal muscle, female kidney, male liver, and in both male and female WAT. In the female small intestine, we specifically analyzed PSMC1 and PSMC2. Finally, we evaluated all UPP components in male BAT and displayed those with statistically significant findings (Figure 3). 

In skeletal muscle, female rats showed significant downregulation of PSMC4 and PSMC2 at weeks 4 and 8, with all PSMCs showing negative trends. In contrast, the male liver showed upregulation of multiple proteasome subunits (PSMC2, PSMC3, PSMC4, PSMC5) at weeks one and four. WAT from females exhibited a significant decrease in PSMC1 at week one. In the male heart, PRMT1 expression was significantly decreased at both weeks four and eight.

## Discussion

The results of this study provide insights into the complexities of how exercise may impact protein degradation and ADMA/SDMA production in both sexes across multiple tissues. Exercise induced distinct tissue- and sex-specific changes in markers of proteolysis and ADMA/SDMA production. In both male and female rats, the small intestine consistently showed significant reductions in ADMA levels at weeks two, four, and eight, while SDMA levels also declined significantly at weeks two and four in female rats and at week eight in male rats, suggesting this tissue may undergo sustained suppression of proteolysis-related methylarginine production during prolonged training. In contrast, the kidney demonstrated significant increases in SDMA in female rats at week two and in male rats at weeks one, four, and eight. Female kidneys also exhibited increased expression of *PSMC1* and other proteasome-related genes, indicating enhanced proteasome activity. Previous studies have shown that disruption of the UPP is involved in the pathogenesis of various kidney diseases [30]. Thus, more research should confirm if increased activity of proteasome during exercise is involved in the attenuation of the development of kidney pathologies.

Systemic and adipose-specific transcriptional changes further support tissue-specific effects on proteolytic patterns. Male blood RNA showed significant downregulation of nearly all *PRMT* genes at week eight, possibly reflecting a systemic suppression of methyltransferase activity. Meanwhile, in male BAT, PRMT 1-3, enzymes responsible for ADMA synthesis, were upregulated, suggesting localized enhancement of ADMA production. These findings highlight the complexity of exercise-induced regulation of proteolysis and methylation pathways, with divergent responses across tissues and between sexes. Changes in global proteomics for PRMTs and PSMCs following training were mostly statistically insignificant. However, some notable significant changes included increases in male liver PSMCs, decreases in female skeletal muscle PSMCs, and decreases in male heart PRMT1. 

Comparing these findings to previous literature provides important context. Prior studies have established that exercise increases skeletal muscle proteolysis, followed by an adaptive rise in protein synthesis to support muscle remodeling [7,8]. Similarly, previous whole-body measurements of protein turnover have shown a net increase in proteolysis during exercise [9,10]. However, our study extends these findings beyond muscle, demonstrating that training-induced proteolysis may also occur in the kidney along with decreases in other tissues, albeit to varying extents. Since we show that exercise does have an effect on protein metabolism, our results may also support prior research indicating that a sedentary lifestyle disrupts homeostasis of protein metabolism in multiple tissues [12]. Studies have reported that obesity alters muscle protein turnover and impairs the muscle protein synthetic response to exercise [12,13]. Furthermore, previous studies on hepatic proteolysis have yielded conflicting results, with some indicating that exercise enhances liver autophagy [14], while others report that the effects of exercise on liver proteolysis vary across species [15]. Our study contributes to this discourse by showing that exercise may have sex-specific responses in the liver.

This study has several limitations that should be considered when interpreting the findings. First, it relies heavily on secondary data from the MoTrPAC dataset rather than primary experimental data. Thus, key details regarding laboratory conditions, sample preparation, sequencing pipelines, and quality-control procedures are based on information provided by previous MoTrPAC studies [2,6]. Second, global proteomics data were incomplete across multiple tissues, restricting comprehensive multi-tissue comparisons and weakening integration of multi-omic findings. These factors affect methodological transparency and replicability. While the analysis can be reproduced using the same dataset, full experimental replication depends on the reporting of omics processing and analytical methods available on the MoTrPAC Data Hub [2]. Another limitation of this study is the reliance on RNA-seq data to infer changes in protein degradation. While RNA-seq provides valuable insights into gene expression patterns, it does not directly measure protein levels or protein turnover rates. Moreover, since the proteomics data are incomplete and RNA-based data are not a direct measure of protein expression, it is challenging to draw conclusions about functional proteolytic outcomes. Thus, our study only suggests an association between exercise and proteolysis-related markers rather than a causal metabolic benefit. Future studies should incorporate more direct measurements of proteolytic activity, such as radiolabeled amino acid tracing or in vivo proteasome activity assays, to confirm these findings. Finally, the use of rodent models, which, while informative, may not fully capture the complexity of human metabolic responses to exercise and obesity.

Overall, our results suggest that exercise may be associated with increases in ADMA/SDMA levels, as well as *PRMT* and *UPP* gene expression, in some tissues, but decreases in others. Thus, it is unlikely that ADMA and SDMA production in various tissues is directly upregulated and downregulated in opposing methods by exercise. Based on these findings, it is possible that exercise promotes ordered, beneficial proteolysis in some tissues but inhibits proteolysis in others. This could be an opposing effect of the damaging inflammatory effects of obesity. However, because of the limitations of our study design, a causal relationship cannot be definitively concluded. 

## Conclusions

Exercise training is associated with complex tissue- and sex-specific remodeling of methylarginine-related metabolites and some proteostasis-related transcripts. These patterns generate hypotheses about altered protein turnover and methylarginine handling, but do not establish causal mechanisms or direct changes in proteolytic flux. These findings have implications for understanding the metabolic consequences of exercise. The observed changes in ADMA/SDMA levels with exercise suggest that protein degradation plays a role in tissue adaptation to physical activity in tissues other than skeletal muscle. This underscores the potential therapeutic benefits of targeted interventions aimed at modulating proteolysis to improve metabolic health. 

Future studies can investigate whether chronic exercise training can mitigate obesity-induced alterations in proteolysis and ADMA/SDMA production. More research should also evaluate the liver and kidneys’ ability to clear ADMA/SDMA under different physiological conditions, providing a more comprehensive understanding of systemic protein turnover regulation. Ultimately, these findings show the potential for future research aimed at understanding the role of proteolysis and ADMA/SDMA in metabolic health.
